# Metformin inhibits gastric cancer cells metastatic traits through suppression of epithelial-mesenchymal transition in a glucose-independent manner

**DOI:** 10.1371/journal.pone.0174486

**Published:** 2017-03-23

**Authors:** Shiva Valaee, Mohammad Mehdi Yaghoobi, Mehdi Shamsara

**Affiliations:** 1 Research Department of Biotechnology, Institute of Science and High Technology and Environmental Sciences, Graduate University of Advanced Technology, Kerman, Iran; 2 Department of Animal Biotechnology, National Institute of Genetic Engineering and Biotechnology, Tehran, Iran; University of South Alabama Mitchell Cancer Institute, UNITED STATES

## Abstract

Epithelial-mesenchymal transition (EMT), which is mainly recognized by upregulation of mesenchymal markers and movement of cells, is a critical stage occurred during embryo development and spreading cancerous cells. Metformin is an antidiabetic drug used in treatment of type 2 diabetes. EMT inhibitory effect of metformin has been studied in several cancers; however, it remains unknown in gastric cancer. The aim of the present study was to investigate the metformin effects on inhibition of EMT-related genes as well as migration and invasion of AGS gastric cancer cell line. Moreover, to study the effect of glucose on metformin-mediated EMT inhibition, all experiments were performed in two glucose levels, similar to non-fasting blood sugar (7.8 mM) and hyperglycemic (17.5 mM) conditions. The results showed reduction of mesenchymal markers, including vimentin and β-catenin, and induction of epithelial marker, E-cadherin, by metformin in both glucose concentrations. Furthermore, wound-healing and invasion assays showed a significant decrease in cell migration and invasion after metformin treatment in both glucose levels. In conclusion, our results indicated that metformin strongly inhibited EMT of gastric cancer cells in conditions mimicking normo and hyperglycemic blood sugar.

## Introduction

Gastric cancer is the second leading cause of cancer-related mortality in the world [1]. This high mortality rate arises from that a large number of patients are diagnosed at the advanced stages of gastric cancer when the tumor metastasis has been occurred. While detection of gastric cancer in early stages before its spread generally extends survival rate up to five-year higher.

Although several molecular markers have been associated with the metastatic human cancers, one of the main agents contributing to tumor spread is a phenomenon, which is described as epithelial-mesenchymal transition (EMT) [2]. EMT is naturally occurred during embryonic development, but recently, it has been become one of the especial research efforts in many cancers, including gastric cancer [3–6]. EMT is associated with loss of expression of epithelial cell markers, such as E-cadherin and increased expression of mesenchymal genes, like vimentin [6–8]. Vimentin is an intermediate filament protein transcribed by wnt/β-catenin signaling pathway in mesenchymal cells as well as in several types of invasive human epithelial carcinomas [9]. Principal role of β-catenin has been indicated in progression of variety carcinoma and in cancer metastasis through facilitating EMT process [10, 11].

Transforming cells reprogram their normal metabolic functions to provide ATP and enough energy for other biosynthetic processes [12, 13]. Generally, the glucose level of cell culture medium is 25 mM (450 mg/dL), while the fasting blood glucose level in normal condition is between 4–6 mM (around 72–108 mg/dL) and reaches to 7.8 mM (140 mg/dL) 2 hours after eating. Since the glucose has a pivotal role in cancer cells proliferation, targeting of tumor metabolism by reducing accessible glucose has been applied as a novel strategy for cancer treatment [13]. Metformin is a useful drug with limited side effects for treatment of type 2 diabetes by regulating glucose and fatty acid metabolism [14]. It has been also introduced as a novel anticancer agent regulating cell cycle through activation of AMP-activated protein kinase (AMPK) as well as through its effects on tyrosine kinases and insulin absorption [6, 15, 16]. While the clear mechanism for cancer suppression by metformin remains unidentified, metformin systemic actions is likely coordinated with the direct cellular action to promote its effect on cancer growth[17].

Previous study showed that metformin inhibits gastric cancer via the inhibition of HIF1α/PKM2 signaling pathway [18]. However, its anti-metastatic effects and/or regulating EMT remain as an unresolved issue in gastric cancer. Here, we showed that metformin was a potential inhibitor of EMT in AGS gastric cancer cell line and its effect was potentiated by the time, but independent from glucose concentration of medium.

## Materials and methods

### Reagents

Metformin was purchased from Santa Cruz. Thiazolyl blue tetrazolium bromide was from Sigma-Aldrich. Culturex 96 well BME invasion assay kit was purchased from R&D systems. PrimeScript™ RT Master Mix and SYBR Premix Ex Taq reagents were purchased from Takara Biotechnology. The human AGS gastric cancer cell line was purchased from the National Cell Bank of Iran. DMEM/F12 medium and fetal bovine serum (FBS) were purchased from Gibco. TriPure isolation reagent was from Roche. Enhanced chemiluminescence reagent (ECL) was purchased from Najm Biotech. Antibodies against vimentin (sc-32322), β-catenin (sc-7963), E-cadherin (sc-7870) were purchased from Santa Cruz. GAPDH antibody (G9545) was from Sigma. HRP-linked anti-mouse (ab6789) and anti-rabbit (ab97051) IgG were purchased from Abcam.

### Cell line

The human AGS gastric cancer cell line was cultured in DMEM/F12 medium supplemented with 10% FBS and 1% penicillin-streptomycin at 37°C in a humidified atmosphere of 95% air plus 5% CO_2_. Glucose effects on anti-EMT properties of metformin were studied by adjusting the medium glucose at the levels of 7.8 and 17.5 mM, which designated as low- and high-glucose conditions, respectively.

### MTT assay

The cells were seeded into 96-well plates at a density of 5 × 10^3^ cells/well in two different glucose conditions. Next day, the cells were treated with concentrations of metformin (0, 2.5, 5, 10, 20, 50, 100 mM) in triplicate or with PBS in controls and incubated up to 72 hours. Time-dependent effect of metformin was assayed by MTT test at 24-hour intervals for three days. Briefly, cell viability was tested by adding 10 μL of 5 mg/mL tetrazolium salt solution to the medium of each well and incubated at 37°C for 4 hours. After removing the medium, formazan crystals were dissolved in 100 μL DMSO and the absorbance was read at 570 nm (Awareness Technology). The absolute values of the absorbance were converted to surviving fraction data and reported as the percentage of living cells in relation to control. The minimal inhibitory concentration (MIC) of metformin on cell growth was chosen for the next experiments.

### Cell migration test

The cells were seeded in 6-well plates and cultured in the two different glucose conditions to grow as confluent monolayer. A line was created by scraping the cells with a sterile pipette in the middle of the well. The cells were then treated with the MIC of metformin obtained by MTT (10 mM) and the culture was continued at the low-glucose and high-glucose media. The wound area was marked and cell migration into the scratched area was daily photographed using an inverted microscope equipped with camera (Nikon) for two days.

### Cell invasion assay

One day prior to beginning the assay, the AGS cells was starved in a serum-free medium. Invasion assay was performed using the Culturex 96 well BME invasion assay kit. Briefly, top chamber was coated with 0.5 X BME solution at 37°C in a CO_2_ incubator for 4 hours and then plated with 5 × 10^4^ cells in serum-free DMEM/F12 medium with 10mM metformin. Bottom chamber was filled with medium containing FBS and incubated at 37°C with 5% CO_2_ for 24 hours. After washing the chambers, diluted Calcein–AM stock solution was added into the bottom chamber and photographed by fluorescence microscope. The total numbers of invaded cells were counted by imageJ software (NCBI). All experiments were independently repeated at least three times.

### RNA isolation and reverse transcription

The AGS cells were treated with metformin in low-glucose and high-glucose conditions for three days. Total RNA was extracted from the treated cells every 24 hours for three days using TriPure isolation reagent according to the manufacture’s instruction. The RNA was subjected to cDNA synthesis using a PrimeScript^TM^ RT reagent kit. Briefly, the reactions were prepared at 10 μL volume containing1 μg the extracted RNA, 0.5 μL random hexamers, 0.5 μL oligo-dT, 0.5 μL reverse transcriptase and RNase free water and incubated at 37°C for 15 min and then at 85°C for 5 sec.

### Quantitative polymerase chain reaction (qPCR)

The real-time qPCR was performed in the Rotor-Gene 6000 instrument(Corbett) by the SYBR® Premix Ex Taq^TM^ II kit using the thermal cycles of one step 95°C for 30 sec, 40 cycles of 95°C for 5 sec and 58°C for 30 sec. The data were normalized against GAPDH transcript as a reference gene and levels of RNA expression were determined with the 2^-∆∆*Ct*^ method. The sequences of primers are presented in Table 1.

**Table 1:** The primers sequences used in this study.

Name	Sequence (5´ to 3´)	PCR size
GAPDH (NM_001289745.1)	TGGGCTACACTGAGCACCAG	72 bp
	CAGCGTCAAAGGTGGAGGAG	
β-catenin (NM_001904)	ATGGGTGGGACACAGCAGCA	92 bp
	ATGGGTGGGACACAGCAGCA	
Vimentin (NM_003380.3)	CGGGAGAAATTGCAGGAGGAG	106 bp
	CAAGGTCAAGACGTGCCAGAG	
E-cadherin (NM_004360.3)	CGCTTACACCATCCTCAGCCA	118 bp
	AGGGAAACTCTCTCGGTCCAG	

### Western blotting

Total protein was extracted from the controls and metformin-treated cells cultured in two different glucose conditions by RIPA (radioimmuno-precipitation assay) buffer (50 mM Tris, 0.15 M NaCl, 1 mM EGTA, 1% NP40, 0.25% SDS, pH 7.4). The cell lysate was quantified for protein content using Bradford assay. Equal amounts of the cell lysate (50 μg) were separated on 12% SDS-polyacrylamide gel and transferred onto a polyvinylidene fluoride membrane (PVDF; Roche Diagnostics). The membrane was blocked with 5% BSA in Tris-buffered saline containing 0.1% Tween-20 (TBS-T) for one hour at room temperature. The membrane was then incubated with the appropriate antibody in TBS-T with 3% BSA for or overnight at 4°C. After washing in TBS-T, the membrane was incubated with the appropriate horseradish peroxidase (HRP)-conjugated secondary antibody. The proteins were detected using the ECL reagent. The used antibodies were diluted as follow: GAPDH antibody at 1:10,000 dilution, vimentin, β-catenin, E-cadherin antibodies at 1:500 dilutions, HRP-linked anti-mouse and anti-rabbit IgG at 1:2000 dilutions.

### Statistical analysis

All experiments were repeated three times and the statistical analyses were performed using ANOVA or the Student's t-test. The *p*-values < 0.05 were considered to indicate statistically significant differences. Additionally, JMP software was used to investigate the significance of each parameter, including metformin and glucose, separately on EMT. This one achieved by introduced invasion and migration data as a fixed factor, biological replicates, treatment and glucose concentration as random factors. ANOVA was then followed by Tukey’s multiple range test (*p* < 0.05) in order to identify glucose concentration different effects.

## Results

### Cell growth inhibitory effects of metformin

**Fig 1:**
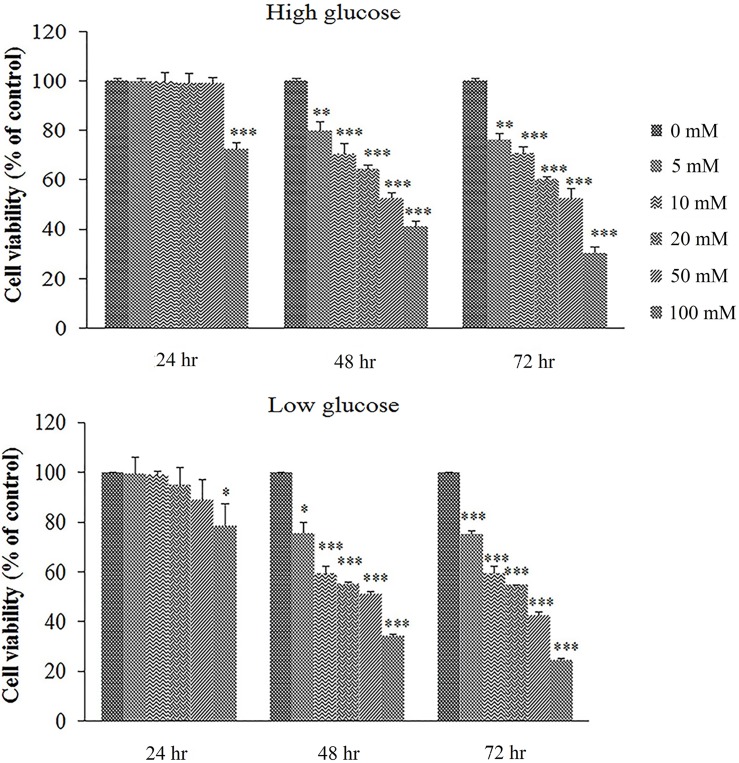
Cell toxicity of metformin in two glucose concentrations. Metformin was used at 0–100 mM in low- and high-glucose media and its inhibitory effects on cells were measured with MTT assay. Each column shows the mean and SD of three independent experiments, performed in triplicate. *Significantly different from untreated cells (*p ≤ 0.01, **p ≤ 0.001 and ***p ≤ 0.0001).

Minimal dose of metformin inhibiting the growth of AGS cells was calculated by MTT test. To reach this goal, metformin was added at the concentrations of 0–100 mM in low- and high-glucose media, separately. To study time-dependent effects of metformin on cell growth, MTT was performed at 24, 48 and 72 hours after treatment. As shown in Fig 1, the MIC of metformin on cell growth was determined to be 10 mM after 48 hours treatment in low- and high-glucose media. With AGS cell, change in morphology was not observed in metformin-treated cells in low- and high-glucose conditions. Metformin almost at the all used concentrations suppressed cell growth at the second day of treatment in both glucose conditions. However, metformin at the high concentration of 100 mM inhibited the cell growth from the first day of treatment. These data showed that metformin inhibited cell growth in dose- and time-dependent routes.

### Metformin effect on EMT markers

**Fig 2:**
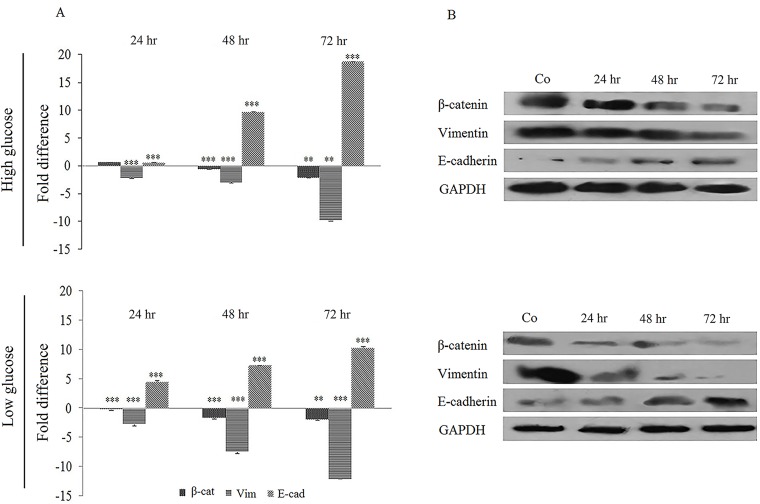
Metformin effects on EMT markers in two glucose levels. (A) The cells were treated with metformin for 72 hours in two glucose concentrations and the desired genes were quantified by real-time qPCR. For normalization, GAPDH was amplified in each sample. Each column shows the mean and SD of three independent experiments, performed in triplicate. (B) Western blot analysis of cells treated with 10 mM metformin for 24 to 72 hours in two different glucose concentrations. The GAPDH band, which is considered as control, confirms the integrity and equal loading of protein.

The change of EMT markers expression in metformin-treated cells was quantified at mRNA level by real-time PCR. As shown in Fig 2A, E-cadherin was upregulated to 0.5, 9.69 and 18.69-fold in high-glucose medium and to 4.45, 7.03 and 10.28-fold in low-glucose one after 24, 48 and 72 hours treatment with metformin, respectively. In the same time, however, vimentin showed a decreased expression to -2.12, -2.96 and -9.78-fold in high-glucose medium and to -2.71, -7.45 and -12.04-fold in low-glucose medium. β-catenin was also down-regulated to 0.62, -0.61, -2.18-fold in high glucose culture and to -0.19, -1.66, -1.92-fold in low glucose culture in the same period. These data showed a time-dependent alteration pattern of gene expression due to metformin treatment.

For further study, the expression of desired genes was quantified at protein level. Western blot results with appropriate antibodies was in consistence with the real-time qPCR data. Vimentin and β-catenin proteins both decreased in response to metformin treatment, while the level of E-cadherin was increased (Fig 2B). The protein change pattern was similar in both glucose culture media.

### Metformin inhibits cell migration and invasion

**Fig 3:**
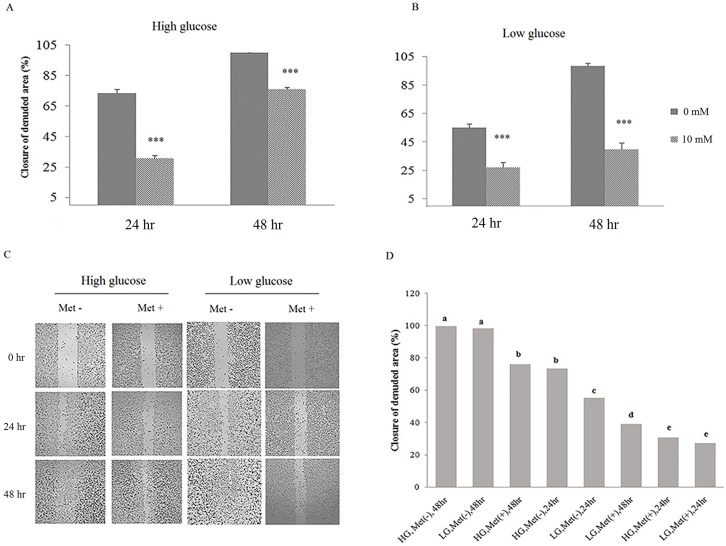
Glucose effects on metformin-inhibited cell migration. (A-C) AGS cells were treated with metformin for 48 hours. Cell migration was quantified by wound-healing assay using ImageJ software (*p < 0.01, **p ≤ 0.001 and ***p ≤ 0.0001). (D) Effect of glucose concentration on cell migration was analyzed by JMP software and levels not connected by same letter are significantly different.

EMT is characterized by loss of cell-to-cell adhesion and increased rate of cell migration and metastasis. For studying the adverse effects of metformin on EMT, cell migration and invasion were assayed by wound-healing and transwell methods. The AGS cells were cultured until become confluent, scratched for creation of a wound and then healing step was studied in treated and untreated (control) cultures for two days. Wound healing progress in control culture was rapid such that the scratch was disappeared in 48 hours, while in treated samples, metformin inhibited the healing process and the closure of the denuded area progressed more slowly (Fig 3A–3C). Comparing the wound-healing results in two glucose concentrations indicated that metformin inhibition of cell migration was not affected by glucose (Fig 3D).

**Fig 4:**
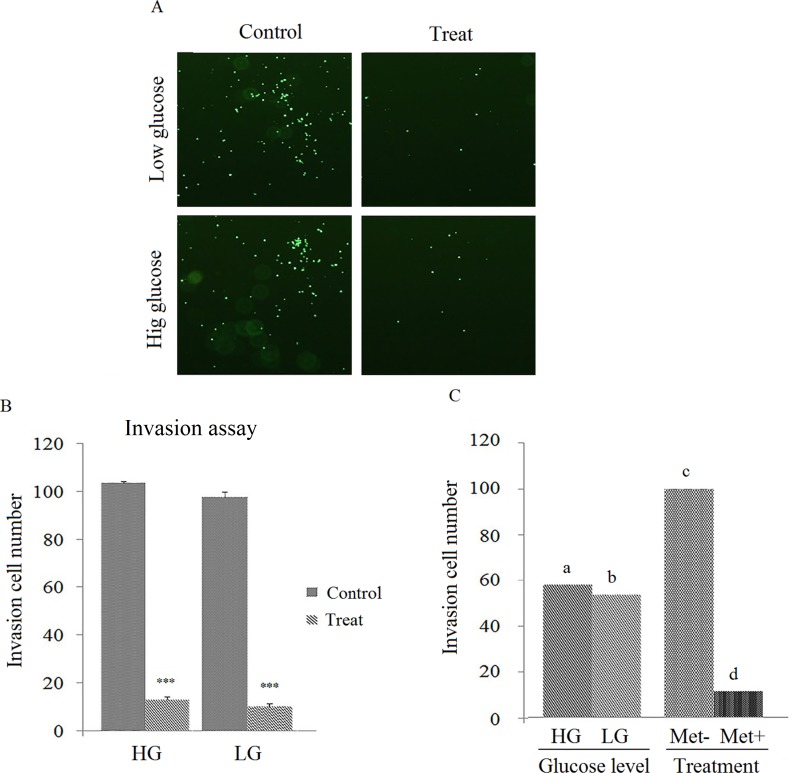
Glucose effects on metformin-inhibited cell invasion. **(**A & B) AGS cells were treated with metformin for 24 hours and the cell invasion was measured in the Culturex 96 well BME invasion assay kit by counting the number of cells invading underside of BME (*p ≤ 0.01, **p ≤ 0.001 and ***p ≤ 0.0001). (C) Effect of high- and low-glucose media on cell invasion analyzed by JMP and levels not connected by same letter are significantly different (*p* ≤ 0.05).

After plating the cells into the transwell chamber, the cell migration ability was measured after 24 hours. As shown in the Fig 4A & 4B, the metformin markedly decreased the invasion ability of the AGS cells. Additionally, the glucose concentration has no significant effect on metformin ability to inhibit cell invasion (Fig 4C).

## Discussion

Upregulation of EMT markers in cancers play a critical role in cell invasion and cancer metastasis [19–22]. Hence, EMT inhibition is considered as a pivotal therapeutic step in cancers treatment. A number of studies refer to anti-proliferation and anti-metastatic effects of metformin on several cancers [23, 24]. However, the adverse effects of metformin on gastric cancer remain obscure. The aim of the present study was to investigate the effect of glucose levels on metformin-mediated inhibition of EMT in human gastric cell line. For this purpose, the AGS cells were cultured in glucose concentrations of 7.8 and 17.5 mM and treated with 10 mM metformin. We found that metformin suppressed cell migration and invasion in both glucose conditions in a time-dependent manner, such that the strongest repression was observed in 72-hour treatments. Further analyses revealed the induction of E-cadherin (epithelial marker) and the reduction of β-catenin and vimentin (EMT markers). Furthermore, the metformin anti-EMT activity was similar in the both glucose concentrations.

Metformin inhibits EMT at the range of 2.5–50 mM in different cell lines [6, 17, 18, 25, 26]. We used 10 mM metformin in our study, which is around 200-fold higher than the plasma level of metformin in type 2 diabetic patients [27]. It seems that reaching to such levels of metformin at *in vivo* is impossible and even may be toxic for physiologic condition. However, high concentrations of FBS and other nutrients in the culture medium are powerful stimulators of cell growth. Hence, a higher dose of metformin must be used to inhibit EMT behavior of cells in *in vitro* condition. Another difference between the *in vitro* and *in vivo* systems is the duration time that the experiments need to be completed. While the cell culture experiments are finalized in a few days, the *in vivo* studies need to a long time, sometimes up to several months or even years, to complete. Long time exposure to metformin causes the appearance of drug therapeutic effects at low concentrations. Moreover, it has been demonstrated that metformin accumulates in tissues several folds higher than in the blood and in the mitochondrial matrix it could accumulate until 1000-fold (> 20 mmol/L) [27–29]. Taken together, these indicate that cell culture results of EMT inhibition by metformin are applicable for *in vivo* conditions.

Our study indicated that metformin treatment time-dependently led to increase of E-cadherin in the gastric cancer cells. E-cadherin is a well-known cell-cell adhesion molecule forming epithelial adherent junctions. Loss of E-cadherin expression is one of the most predominant hallmark of EMT occurring during the progression of many cancers [30–32]. According to Liu and et al. study [33], E-cadherin down-regulation in gastric cancer positively enhanced cell survival and metastasis through Wnt/β-catenin signaling pathway and reversing this process might be a useful subject for clinical features of gastric cancer. Furthermore, promoting role of E-cadherin in tumor regression of epithelial cancers has been proposed by the previous studies [34–36]. We showed that EMT inhibition by metformin in the gastric cancer cell line was in concordant with E-cadherin enhancement. This is important because of pivotal role of E-cadherin in regression of metastatic behavior of epithelial cancers, such as gastric cancer.

Metformin-mediated suppression of vimentin has been described previously [37]. In line with these reports, we obtained similar results in gastric cancer cells. Vimentin is the most well-known hallmark of mesenchymal phenotype in cancers, which is upregulated through β-catenin/TCF complex. Vimentin dysregulation possess significant impacts on tumor growth, cellular motility, and poor prognosis [38, 39]. Fuyuhire et al. indicated that there is a direct relationship between vimentin expression and advanced clinical stage in gastric cancer [22]. In addition, vimentin interacts with mitochondrial outer membrane to control respiratory chain activity and ATP production [40]. Upregulated Vimentin stimulates cell migration through enhancement of mitochondrial membrane potential [41, 42]. The essential role of upregulated vimentin in invasion of gastric cancer shows the importance of our finding in vimentin suppression by metformin.

Consumption rate of glucose by cancer cells is higher than that of normal cells. Glucose is necessary for the proliferation of cancer cells and therefore, reduction of available glucose can be used as an anti-cancer treatment strategy acting through targeting tumor metabolism [13]. For example, proliferation of Jurkat leukemia cells seeded in 1 mM glucose rapidly ceased as the glucose exhausted [43]. Therefore, low-glucose availability maybe improves the anti-proliferative and anti-metastatic activity of metformin. We examined this hypothesis, but our finding indicated the similar cytotoxicity of metformin on the gastric cancer cells in low- and high-glucose conditions. Inconsistence with our results, Zhuang et al. [17] reported the improvement of metformin cytotoxicity on breast and ovarian cancers progress in limited glucose condition. They described that metformin treatment in glucose-starved condition led to decrease of ATP in cancer cells. While, at higher glucose concentration, metformin compensated the abolished level of ATP by enhancement of glycolysis through activation of AMP-activated protein kinase. This inconsistency maybe raised in part by the different glucose level used as low-glucose state in these studies. Glucose amount of culture media currently is 25 mM (450 mg/dL), while normal blood sugar is between 4 to 6 mM (around 72–108 mg/dL) and reaches to 7.8 mM (140 mg/dL) 2 hours after eating. In hyperglycemic condition, like diabetes, it reaches to 17.5 mM. While, we designated non-fasting glucose level (7.8 mM) as low-glucose state, Zhuang study used 2.5 mM glucose, a level which rarely occurs during starvation state in the body. Indeed, the consumption of metformin at such low level of blood sugar maybe result in dangerous or even lethal condition for patients, like lactic acidosis. Based on the present study, we indicated similar anti-cancer activity of metformin on gastric cancer cells in the media mimicking the non-fasting and hyperglycemic blood sugar. This finding might be shows that anti-cancer activity of metformin in diabetic patients is as much as non-diabetic patients.

In summary, we indicated that metformin strongly inhibited invasion and migration of gastric cancer cells through EMT suppression. A time-dependent regression of EMT markers was achieved by metformin, as the most inhibition was observed at 72-hours treatments. In addition, the similar effect of metformin on gastric cancer cell line growing the cells in conditions relevant to normal non-fasting and hyperglycemic blood sugar concluded that metformin might be possessed the same anti-cancer activity in diabetic and non-diabetic patients. Of course, further studies are required to confirm these findings in other gastric cancer cell lines.
